# Does the Effect of PM_10_ on Mortality Depend on PM Nickel and Vanadium Content? A Reanalysis of the NMMAPS Data

**DOI:** 10.1289/ehp.10737

**Published:** 2007-09-25

**Authors:** Francesca Dominici, Roger D. Peng, Keita Ebisu, Scott L. Zeger, Jonathan M. Samet, Michelle L. Bell

**Affiliations:** 1 Department of Biostatistics, Johns Hopkins Bloomberg School of Public Health, Baltimore, Maryland, USA; 2 School of Forestry and Environmental Studies, Yale University, New Haven, Connecticut, USA; 3 Department of Epidemiology, Johns Hopkins Bloomberg School of Public Health, Baltimore, Maryland, USA

**Keywords:** effect modification, mortality, Ni, particulate matter, PM_2.5_, PM_10_, V

## Abstract

**Background:**

Lack of knowledge regarding particulate matter (PM) characteristics associated with toxicity is a crucial research gap. Short-term effects of PM can vary by location, possibly reflecting regional differences in mixtures. A report by Lippmann et al. [Lippmann et al., Environ Health Perspect 114:1662–1669 (2006)] analyzed mortality effect estimates from the National Morbidity, Mortality, and Air Pollution Study (NMMAPS) for 1987–1994. They found that average concentrations of nickel or vanadium in PM_2.5_ (PM with aerodynamic diameter < 2.5 μm) positively modified the lag-1 day association between PM_10_ and all-cause mortality.

**Objective:**

We reestimated the relationship between county-specific lag-1 PM_10_ (PM with aerodynamic diameter < 10 μm) effects on mortality and county-specific nickel or vanadium PM_2.5_ average concentrations using 1987–2000 effect estimates. We explored whether such modification is sensitive to outliers.

**Methods:**

We estimated long-term average county-level nickel and vanadium PM_2.5_ concentrations for 2000–2005 for 72 U.S. counties representing 69 communities. We fitted Bayesian hierarchical regression models to investigate whether county-specific short-term effects of PM_10_ on mortality are modified by long-term county-specific nickel or vanadium PM_2.5_ concentrations. We conducted sensitivity analyses by excluding individual communities and considering log-transformed data.

**Results:**

Our results were consistent with those of Lippmann et al. However, we found that when counties included in the NMMAPS New York community were excluded from the sensitivity analysis, the evidence of effect modification of nickel or vanadium on the short-term effects of PM_10_ mortality was much weaker and no longer statistically significant.

**Conclusions:**

Our analysis does not contradict the hypothesis that nickel or vanadium may increase the risk of PM to human health, but it highlights the sensitivity of findings to particularly influential observations.

Associations between particulate matter (PM) total mass for PM_10_ (PM with an aerodynamic diameter < 10 μm) and PM_2.5_ (PM with an aerodynamic diameter < 2.5 μm) have been demonstrated for mortality and numerous morbidity outcomes including hospital admissions and pulmonary function [Dominici et al. 2006; Pope and Dockery 2006; U.S. Environmental Protection Agency (EPA) 2004]. However, the effect estimates vary by location and season, as shown by recent national studies of PM_2.5_ and hospital admissions (Dominici et al. 2006) and PM_10_ and mortality (Peng et al. 2005). The chemical composition of the particulate mixture also exhibits substantial regional and seasonal variation (Bell et al. 2007); this variation may contribute to the heterogeneity in PM health effect estimates.

Lippmann and colleagues (2006) recently conducted an investigation on chemical components of ambient PM_2.5_ and mortality risk. In addition to reporting findings of an inhalation exposure study in mice, the authors examined whether previously calculated effect estimates for PM_10_ on mortality from the National Morbidity, Mortality, and Air Pollution Study (NMMAPS) (Dominici et al. 2003; Samet et al. 2000a, 2000b) were associated with the communities’ long-term levels of various PM_2.5_ chemical components. They estimated the association between the short-term effects of PM_10_ on all-cause mortality (β̂*^c^*) and average concentrations of 16 PM_2.5_ chemical components (*x^c^*) across 60 U.S. communities. Separately for each chemical component, they fitted a weighted linear regression having β̂*^c^* as the dependent variable and *x^c^* as the independent variable with “weights based on standard errors of the β̂*^c^*,” as reported by the authors (Lippman et al. 2006). The β̂*^c^* and their standard errors were obtained from the NMMAPS data base [Internet-based Health & Air Pollution Surveillance System (iHAPSS) 2007; Zeger et al. 2006] for the period 1987–1994, and the *x^c^* values were obtained from the PM_2.5_ speciation network for the period 2000–2003 (U.S. EPA 2003). Lippman et al. (2006) found that average concentrations of nickel or vanadium PM_2.5_ positively modified the association between the previous day’s (lag 1) PM_10_ and all-cause mortality. Based on this result, the authors concluded that “the PM_10_ mortality risk estimates were high for Ni and V in the communities where Ni and V were significantly high (95th percentile), compared with the communities where Ni and V were low (5th percentile)” (Lippmann et al. 2006). Earlier work identified the previous day as the single-day lag with the strongest effect, and this lag was applied in the work by Lippmann et al. (2006).

We report an analysis similar to that of Lippmann et al. (2006), but using the NMMAPS extended data base for 1987–2000. Our goal was to test whether there is still evidence to indicate that average concentrations of Ni or V PM_2.5_ positively modified the association between the previous day’s (lag 1) PM_10_ and all-cause mortality and whether this evidence is robust to transformation of the data and exclusion of outliers.

## Materials and Methods

As we continue to update NMMAPS data, we have reestimated the short-term effects of PM_10_ on all-cause and cause-specific mortality based on data for 1987–2000 for 90 U.S. urban communities (Dominici et al. 2007; iHAPPS 2007). Further, we have developed a database of PM_2.5_ chemical composition for 2000–2005 for 187 U.S. counties generated by the U.S. EPA (Bell et al. 2007). References to Ni or V in this article reflect the concentrations of those components in PM_2.5_.

Each NMMAPS community is based on a single county or a set of contiguous counties. We have identified 72 U.S. counties that have both an NMMAPS PM_10_ mortality effect estimate and data on PM_2.5_ chemical composition data. These 72 counties are included in 69 NMMAPS communities. More specifically, all NMMAPS communities included in this analysis were based on single counties except two communities. The first is the New York, New York, NMMAPS community, which includes six counties (Bronx, Kings, New York, Richmond, Queens, and Westchester). These counties represent the New York metropolitan area, not the official designation of New York City. PM_2.5_ chemical composition data were available for three counties in the New York community (Queens, New York, and Bronx counties). The second is the NMMAPS Minneapolis community, which includes two counties (Ramsey and Hennepin). PM_2.5_ chemical composition data were available for both counties in the NMMAPS Minneapolis community. For the three counties within the NMMAPS New York community and for the two counties within the NMMAPS Minneapolis community, we used the same value of the NMMAPS effect estimates, respectively.

We estimated the association between the true lag-1 day PM_10_ mortality effect (β*^c^*) and county-level averages of Ni and V (*x^c^*) using the following Bayesian hierarchical regression model:


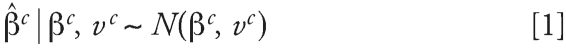



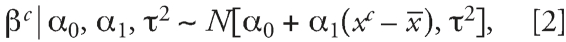


where β̂*^c^* is the NMMAPS community-specific estimate of the effect of lag-1 PM_10_ on mortality, and *v^c^* its statistical variance (Peng 2007; Peng et al. 2005). The parameter α_0_ denotes the true lag-1 effect of PM_10_ on mortality for a county with *x^c^* = ◯. The parameter α_1_ quantifies the effect modification—that is, the change in the true PM_10_ effect estimate associated with a unit change in county-level averages of Ni or V PM_2.5_ (*x^c^*) with respect to their averages across the counties ◯. The parameter τ^2^ denotes the between-county variability of the true lag-1 day effects of PM_10_ on mortality (β*^c^*), unexplained by *x^c^*.

We fitted the above Bayesian hierarchical regression model using two-level normal independent sampling estimation (TLNise) (Everson and Morris 2000) with noninformative priors. We also performed a weighted regression with weights based on the inverse of the variance, 1/*v^c^*. A weighted regression approach was used in the statistical analysis conducted by Lippmann et al. (2006).

## Results

As in Lippmann et al. (2006), we also found strong evidence of effect modification: Counties with high Ni or V average concentrations have higher effects of PM_10_ on mortality at lag 1. We then conducted sensitivity analyses to investigate whether one or a few counties were contributing more than others toward the strength of the evidence of effect modification.

**Figure 1:**
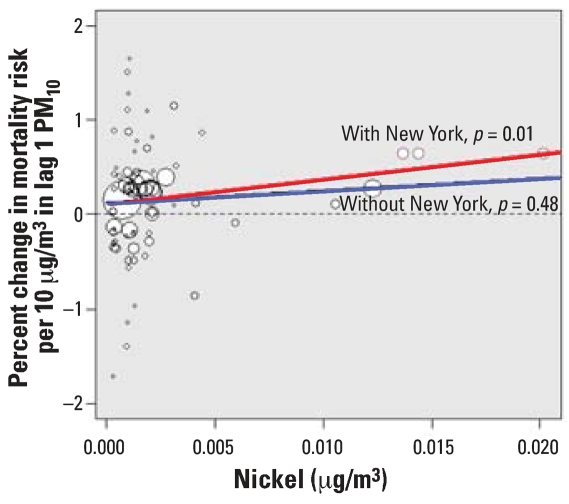
Maximum likelihood estimates of PM_10_ effect on total mortality and county-specific average concentrations of Ni. The size of the circle corresponds to the inverse of the standard error of the community’s maximum likelihood estimate. The red and blue lines denote the fitted linear regression lines with the three counties that belong to the New York community included (red) and excluded from the analysis (blue).

Figure 1 shows the county-specific average concentrations of Ni PM_2.5_ (*x*-axis) plotted against the county-specific maximum likelihood estimates of the lag-1 effects of PM_10_ on mortality (*y*-axis). The size of the circle corresponds to the inverse of the standard error of the county’s maximum likelihood estimate.

**Figure 2:**
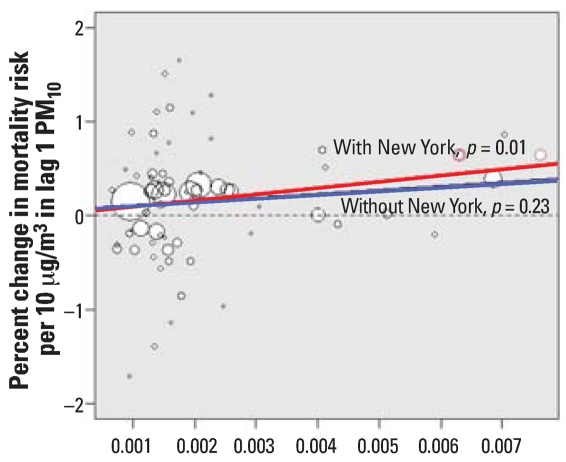
Counties’ maximum likelihood estimates and county-specific average concentrations of V. The size of the circle corresponds to the inverse of the standard error of the community’s maximum likelihood estimate. The red and blue lines denote the fitted linear regression lines with the three counties that belong to the New York community included (red) and excluded from the analysis (blue).

The red and blue lines denote the fitted linear regression lines of the second-stage regression β*^c^* = α0 + α_1_(*x^c^* – ◯) + *N*(0, τ^2^) with the three counties that belong to the NMMAPS New York community included in the analysis (red) and excluded from the analysis (blue). Figure 2 shows an analogous figure with county-specific average concentrations of V used as independent variables.

When all 72 counties were included in the analyses, we found strong evidence of effect modification by either Ni or V. The posterior probability that the parameter α_1_ is positive is 0.99 for Ni and 1.0 for V. The *p*-values corresponding to the statistical significance of α_1_ obtained from the weighted regression are equal to 0.004 and 0.002 for Ni and V, respectively.

When the three counties that belong to the NMMAPS New York community were excluded, evidence of effect modification became much weaker, with loose statistical significance. The posterior probability that the effect modification parameter α_1_ is positive is 0.76 for Ni and 0.89 for V. The *p*-values corresponding to the statistical significance of α_1_ obtained from the weighted regression are 0.38 for Ni and 0.14 for V.

**Figure 3:**
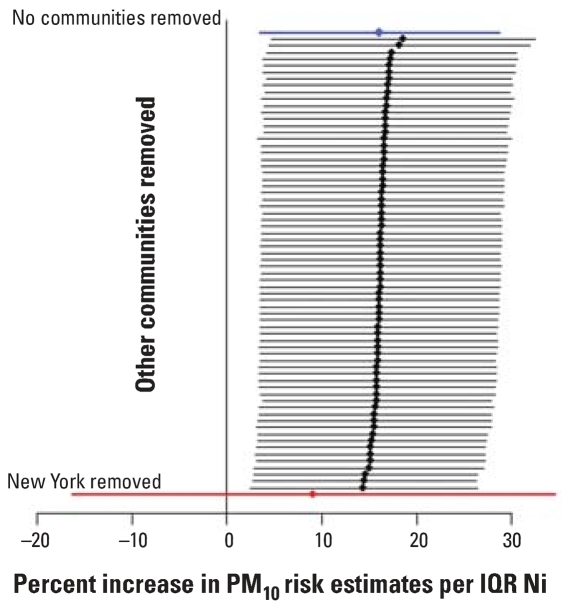
Point estimates and 95% confidence intervals of the percent increase in PM_10_ risk estimates associated with an interquartile range (IQR) increase in Ni PM_2.5_. The top estimate (in blue) is achieved by including data for all the 69 communities. The other estimates are calculated by excluding one of the 69 communities at a time. The last estimate (in red) is obtained when the New York community is excluded.

**Figure 4:**
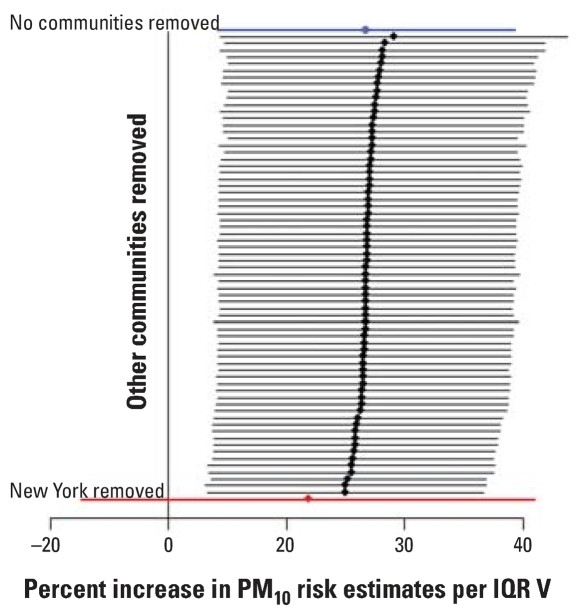
Point estimates and 95% confidence intervals of the percent increase in PM_10_ risk estimates associated with an interquartile range (IQR) increase in V PM_2.5_. The top estimate (in blue) is achieved by including data for all the 69 communities. The other estimates are calculated by excluding one of the 69 communities at a time. The last estimate (in red) is obtained when the New York community is excluded.

To further investigate the sensitivity of the estimated effect modification parameter to outliers, we reestimated α_1_ by excluding a single county at a time, for each of the 72 counties. Figures 3 and 4 show the posterior means and 95% posterior intervals of the effect modification parameter α_1_ obtained by excluding one county at a time for Ni and for V as independent variables, respectively. Again, when the three counties corresponding to the New York community were omitted (shown in red in Figures 3 and 4), we found no evidence of effect modification. However, when any other single county was excluded, strong evidence of effect modification remained.

We reached the same conclusions when we *a*) used the lag-1 NMMAPS estimates of the effects of PM_10_ on mortality for 1987–1994 [the same data used by Lippmann et al. (2006)]; *b*) estimated the effect modification parameter using a weighted linear regression instead of Bayesian hierarchical models; and *c*) used log-transformed data for the independent variables.

## Conclusions

This analysis demonstrates that when the three counties in the NMMAPS New York community are excluded from the analysis, the evidence of effect modification of Ni or V PM_2.5_ on the short-term effects of PM_10_ mortality is much weaker. Setting aside the three counties that belong to the New York community, the between-community variance of Ni is reduced by 68%. Therefore, the statistical power for estimating the slope of the regression line also diminishes substantially.

The New York community has particularly high levels of Ni and V. The three New York counties have the highest Ni concentrations across all the 72 counties. The Ni and V concentrations in the three New York counties were 8.9 and 3.4 times higher than the other counties, respectively,

Elevated levels of Ni and V PM_2.5_ chemical components in New York are likely attributed to oil-fired power plants and emissions from ships using oil, as noted by Lippmann et al. (2006). Ni and V can result from oil combustion and are often used as tracer components for these sources (Chen et al. 2004; Galbreath et al. 2000; Juichang et al. 1995; Thurston et al. 2005; U.S. EPA 2007). Analysis of the sources of PM_2.5_ in New York City identified Ni and V as indicators of oil combustion (Li et al. 2004; Zheng et al. 2004), including ships burning oil as a source (Qin et al. 2006).

Although scientific evidence on the human health impact of PM_2.5_ chemical constituents is limited, several studies have investigated the impacts of Ni or V on health, including an animal study in which V was recovered from lung tissues of rats exposed to concentrated air particles (Morishita et al. 2004). In addition to the analysis of NMMAPS data, Lippmann et al. (2006) found a significant association between exposure to Ni and acute cardiac function changes in mice. Ni and V were associated with urinary 8-OHdG levels, a marker of oxidative DNA damage and repair, and with lower fractional concentration of expired nitric oxide (F_E_NO), a marker of airway responses, in studies of boilermaker workers (Kim et al. 2003, 2004).

Although the original work of Lippmann et al. (2006) indicates strong evidence that the short-term effects of PM_10_ on mortality are modified by long-term averages of Ni and V, our subsequent analysis reveals that this evidence is driven largely by the influence of a few data points (the three New York counties). Our analysis does not contradict the hypothesis that Ni or V may in fact be harmful to human health, but it highlights the sensitivity of findings on effect modification to particularly influential observations.
